# Receipt of Buprenorphine and Naltrexone for Opioid Use Disorder by Race and Ethnicity and Insurance Type

**DOI:** 10.1001/jamanetworkopen.2025.18493

**Published:** 2025-06-26

**Authors:** Utsha G. Khatri, Christopher Lopez, Yun-Ting Yen, Emilia J. Ling, Lynne D. Richardson, Ka Ming Ngai

**Affiliations:** 1 Institute for Health Equity Research, Icahn School of Medicine at Mount Sinai, New York, New York; 2 Department of Population Health Science and Policy, Icahn School of Medicine at Mount Sinai, New York, New York; 3 Department of Emergency Medicine, Icahn School of Medicine at Mount Sinai, New York, New York; 4 Department of Emergency Medicine, Wayne State University, Detroit, Michigan

## Abstract

**Importance:**

Despite expanded efforts to improve treatment access, inequities exist in the receipt of medications for opioid use disorder by race and ethnicity and insurance type.

**Objective:**

To examine inequities in access to medications for opioid use disorder (MOUD)—specifically buprenorphine and naltrexone—by race and ethnicity and insurance type after opioid-related health care events.

**Design, Setting, and Participants:**

This retrospective cohort study used data from the Institute for Health Equity Research Multi-Payor Claims Database, which includes more than 130 million individuals across commercial, Medicaid, and Medicare Advantage insurance plans. The cohort consisted of patients aged 18 years or older with an opioid use disorder (OUD)–related health care event (opioid-related overdose, infection, or treatment event) that occurred between January 1, 2017, and December 31, 2022. Statistical analysis was conducted from October 2023 to December 2024.

**Exposure:**

Race and ethnicity and insurance type, including commercial insurance, Medicaid, and Medicare Advantage, were the primary exposures.

**Main Outcomes and Measures:**

The primary outcome was receipt of MOUD (buprenorphine or naltrexone) within 180 days of an OUD-related health care event. Logistic regression models were used to assess the associations of MOUD receipt with race and ethnicity and with insurance type, adjusting for demographic, clinical, time, and geographical covariates.

**Results:**

Among 176 997 index events involving 164 728 patients between 2017 and 2022, the mean (SD) patient age was 40.0 (13.1) years; 104 005 (58.8%) involved men; 1083 events (0.6%) were among Asian patients, 23 424 (13.2%) were among Black patients, 10 302 (5.8%) were among Hispanic patients, 90 124 (50.9%) were among White patients, and 4697 (2.7%) were among patients of other race and ethnicity; and 147 257 (83.2%) were covered by Medicaid. Black (adjusted estimated probability, 17.1% [95% CI, 13.0%-21.1%]) and Hispanic (adjusted estimated probability, 16.2% [95% CI, 11.6%-20.8%]) patients were significantly less likely than White patients (adjusted estimated probability, 20.5% [95% CI, 16.4%-24.7%]) to receive buprenorphine (Black patients: adjusted odds ratio [AOR], 0.75 [95% CI, 0.63-0.90]; adjusted risk difference [ARD], −3.4 percentage points [pp] [95% CI, –6.5 to –0.4 pp]; Hispanic patients: AOR, 0.69 [95% CI, 0.51-0.92]; ARD, –4.4 pp [95% CI, –9.1 to 0.4 pp]) but received naltrexone at similar rates. Patients with Medicaid (AOR, 1.39 [95% CI, 1.14-1.69]; ARD, 3.5 pp [95% CI, 0.9-6.1 pp]) or Medicare Advantage (AOR, 1.40 [95% CI, 1.05-1.86]; ARD, 3.6 pp [95% CI, –0.6 to 7.7 pp]) were more likely to receive buprenorphine compared with those with commercial insurance. Buprenorphine access improved incrementally from 2017 to 2022, reflecting recent clinical and policy changes; however, disparities persisted.

**Conclusions and Relevance:**

In this cohort study of more than 176 000 opioid-related index health care events, race and ethnicity–based and insurance-based disparities in access to MOUD persisted despite efforts to expand treatment availability. These findings underscore the need for targeted interventions, including culturally tailored care, expanded access points, and policy reforms to address structural barriers and reduce inequities contributing to the overdose crisis.

## Key Points

**Question:**

Are there inequities in access to medications for opioid use disorder (MOUD) by race and ethnicity and insurance type after opioid-related health care events?

**Findings:**

In this cohort study of more than 176 000 opioid-related events from 2017 to 2022, adjusted estimated probabilities showed that Black (17.1%) and Hispanic patients (16.2%) were significantly less likely than White patients (20.5%) to receive buprenorphine. Patients had higher odds of receiving buprenorphine with Medicaid and Medicare Advantage compared with commercial insurance.

**Meaning:**

Despite overall improvements, significant racial and ethnic–based and insurance-based disparities in MOUD access persist and require urgent, targeted solutions.

## Teaser

This cohort study examines inequities by race and ethnicity and insurance type in access to buprenorphine and naltrexone within 180 days after an opioid use disorder (OUD)–related health care event.

## Introduction

The overdose crisis in the US remains a major public health challenge, causing more than 100 000 deaths annually, primarily due to opioids.^1^ Recent data show increasing mortality among members of racial and ethnic minority populations. Between 2021 and 2022, overdose death rates increased for American Indian, Alaska Native, Asian, Black, and Hispanic populations, while decreasing among White populations.^1^ In 2020, the opioid overdose death rate in the US among Black populations surpassed that of White populations for the first time since 1999.^2^ These disparities may be rooted in social and structural inequities, where responses to drug use—ranging from treatment to criminalization—often disproportionately affect marginalized communities.^3,4^

Three medications approved by the US Food and Drug Administration (FDA) for opioid use disorder (OUD)—buprenorphine, naltrexone, and methadone—have been proven to reduce opioid use and mortality, with buprenorphine and methadone (both opioid agonist therapies) having the strongest evidence base.^5^ However, access remains limited. Racial and ethnic disparities persist, with Black and Hispanic individuals less likely to receive medications for OUD (MOUD) than White individuals.^6,7^ Prior studies, often relying on single-payer data, such as Medicaid, Medicare, or commercial insurance claims, have shown that Black and Hispanic patients are substantially less likely to receive buprenorphine—even after accounting for income, geography, and comorbidities.^8,9,10^ This finding suggests that structural factors, such as disparities in pharmacy, prescriber, and program access, may contribute. However, most studies lack longitudinal data or diverse representation across insurance types, especially in light of recent policy changes intended to expand MOUD access.

This study addresses these limitations by using data from the Institute for Health Equity Research Multi-Payor Claims Database, which includes a range of payer types. Spanning from 2017 to 2022, it captures trends during key policy shifts, including expanded telehealth and relaxed buprenorphine prescribing rules during the COVID-19 pandemic.^11,12^ We focused on buprenorphine and naltrexone, 2 MOUD options commonly prescribed in general health care settings and reliably captured in claims data. Unlike methadone, which is limited to highly regulated opioid treatment programs, these medications are available in a broader range of care settings. Analyzing buprenorphine and naltrexone access allows for evaluation of disparities across more accessible treatment environments.

Our primary objective was to assess whether racial and ethnic disparities persisted in buprenorphine and naltrexone receipt in a large, multipayer cohort and to evaluate the association of insurance type with MOUD receipt within 180 days of an OUD-related index event. By using diverse payer data and a large national sample, this study provides a nuanced understanding of how race and ethnicity and insurance type are associated with MOUD access.

## Methods

### Data Sources

This study uses data from the Institute for Health Equity Research Multi-Payor Claims Database, which contains deidentified individual-level data from the HealthVerity Private Source 20 database. The HealthVerity Private Source 20 database includes administrative claims and encounter data from commercial, Medicaid, and Medicare Advantage plans, covering approximately 70 million commercial insurance enrollees, 60 million Medicaid enrollees, and 15 million Medicare Advantage enrollees across 150 payers since 2015. The dataset includes more than 130 million individuals, representing approximately one-third of the US population, and excludes clearinghouse data. This retrospective cohort study followed the Strengthening the Reporting of Observational Studies in Epidemiology (STROBE) reporting guideline and received a waiver of authorization for the use and disclosure of protected health information and a determination of exempt status under 45 CFR §46.104(d)(4) from the Mount Sinai School of Medicine institutional review board. The Mount Sinai School of Medicine institutional review board also granted a waiver of informed consent due to the use of a deidentified limited dataset.

### Study Sample

We conducted a retrospective, longitudinal cohort study of individuals aged 18 years or older with an OUD-related index event between January 1, 2017, and December 31, 2022, across all 50 US states and Washington, DC. Index events included nonfatal opioid overdoses treated in emergency departments or inpatient settings; hospitalizations for injection drug use–related infections (eg, hepatitis C, phlebitis, septic arthritis, skin or soft-tissue infection, or endocarditis) with an OUD diagnosis in the prior 30 days; and inpatient or residential rehabilitation or detoxification care with a primary OUD diagnosis or an OUD diagnosis in the prior 30 days.^10^ To ensure complete data, we restricted the sample to individuals with continuous insurance enrollment for 180 days prior to and 30 days after the index event. The 30-day postindex enrollment criterion was selected to minimize potential bias introduced by coverage instability, particularly among Medicaid beneficiaries, while maximizing cohort representativeness. Although our primary outcome was measured after more than 180 days, we allowed for gaps in coverage after the index event to reflect patterns of insurance churn, especially among individuals with OUD. This approach is consistent with prior literature using similar follow-up windows and enrollment criteria.^9^ Individuals could contribute multiple events if the events were separated by 12 or more months. We excluded patients with long-term prescriptions for opioids (≥90 days’ supply in prior 180 days), as MOUD would likely not be clinically appropriate. We excluded patients with end-stage kidney disease and those receiving hospice care because addiction treatment is not designed to curb end-of-life prescriptions for analgesia (eFigure in Supplement 1).

### Outcome Variables

Our outcome of interest was the receipt of MOUD within 180 days after an index event. We examined the receipt of buprenorphine and/or naltrexone. The receipt of either medication was analyzed descriptively (as shown in Table 1 and Table 2) but was not included as a separate combined outcome in the regression models. As our focus was on community-accessible MOUD, we excluded methadone, which is administered through highly regulated opioid treatment programs and not consistently recorded in outpatient claims. Methadone coverage also varies widely across commercial plans, and its availability differs regionally, limiting its inclusion.

**Table 1.** Characteristics of Beneficiaries and Index Events, 2017-2022

Characteristic	Events, No. (%) (N = 176 997)
Age, mean (SD), y	40.0 (13.1)
Age group, y	
18-24	17 261 (9.8)
25-34	55 842 (31.5)
35-44	44 442 (25.1)
45-64	53 302 (30.1)
≥65	6150 (3.5)
Sex	
Female	72 992 (41.2)
Male	104 005 (58.8)
Race and ethnicity	
Asian	1083 (0.6)
Black	23 424 (13.2)
Hispanic	10 302 (5.8)
White	90 124 (50.9)
Other^a^	4697 (2.7)
Unknown	47 367 (26.8)
Index event type^b^	
Nonfatal overdose	125 987 (71.2)
Rehabilitation or detoxification care	18 996 (10.7)
Injection drug use–related infection	32 014 (18.1)
Index events per person, mean (SD), No.	1.0 (0.3)
Insurance	
Commercial	19 179 (10.8)
Medicaid	147 257 (83.2)
Medicare Advantage	10 192 (5.8)
Other	369 (0.2)
Comorbidities, No.	
None	87 420 (49.4)
1-2	67 002 (37.9)
≥3	22 575 (12.8)
Year	
2017	25 809 (14.6)
2018	24 190 (13.7)
2019	25 204 (14.2)
2020	30 506 (17.2)
2021	36 625 (20.7)
2022	34 663 (19.6)
Medication received within 180 d	
Buprenorphine	34 088 (19.3)
Naltrexone	6491 (3.7)
Either	38 953 (22.0)

^a^ Includes American Indian or Alaska Native and Pacific Islander.

^b^ A nonfatal opioid overdose treated in an emergency department or inpatient setting; hospitalization for an injection drug use–related infection (such as acute hepatitis C, phlebitis, septic arthritis, skin or soft tissue infection, or endocarditis) combined with a diagnosis of opioid use disorder within the past 30 days; or inpatient or residential rehabilitation or detoxification care with a primary diagnosis of opioid use disorder or a diagnosis of opioid use disorder received within the previous 30 days.

**Table 2.** Characteristics of MOUD-Related Index Events by Race and Ethnicity, 2017-2022

Characteristic	Events, No. (%)						
	Total (N = 176 997)	Asian (n = 1083)	Black (n = 23 424)	Hispanic (n = 10 302)	White (n = 90 124)	Other (n = 4697)^a^	Unknown (n = 47367)
Age, mean (SD), y	40.0 (13.1)	38.5 (13.7)	46.9 (13.7)	39.5 (13.4)	38.2 (11.9)	41.0 (13.6)	40.0 (13.8)
Sex							
Female	72 992 (41.2)	340 (31.4)	7914 (33.8)	3230 (31.4)	41 287 (45.8)	1717 (36.6)	18 504 (39.1)
Male	104 005 (58.8)	743 (68.6)	15 510 (66.2)	7072 (68.6)	48 837 (54.2)	2980 (63.4)	28 863 (60.9)
Index event type^b^							
Nonfatal overdose	125 987 (71.2)	799 (73.8)	18 474 (78.9)	7771 (75.4)	60 541 (67.2)	3536 (75.3)	34 866 (73.6)
Rehabilitation or detoxification care	18 996 (10.7)	105 (9.7)	1625 (6.9)	598 (5.8)	12 210 (13.5)	304 (6.5)	4154 (8.8)
Injection drug use–related infection	32 014 (18.1)	179 (16.5)	3325 (14.2)	1933 (18.8)	17 373 (19.3)	857 (18.2)	8347 (17.6)
Index events per person, mean (SD), No.	1.0 (0.3)	1.1 (0.2)	1.1 (0.3)	1.1 (0.3)	1.1 (0.3)	1.1 (0.3)	1.1 (0.3)
Insurance							
Commercial	19 179 (10.8)	126 (11.6)	590 (2.5)	522 (5.1)	5754 (6.4)	985 (21.0)	11 202 (23.6)
Medicaid	147 257 (83.2)	893 (82.5)	20 850 (89.0)	9192 (89.2)	79 978 (88.7)	3482 (74.1)	32 862 (69.4)
Medicare Advantage	10 192 (5.8)	61 (5.6)	1942 (8.3)	574 (5.6)	4242 (4.7)	220 (4.7)	3153 (6.7)
Other	369 (0.2)	3 (0.3)	42 (0.2)	14 (0.1)	150 (0.2)	10 (0.2)	150 (0.3)
Medication received within 180 d							
Buprenorphine	34 088 (19.3)	161 (14.9)	2881 (12.3)	1456 (14.1)	20 880 (23.2)	706 (15.0)	8004 (16.9)
Naltrexone	6491 (3.7)	32 (3.0)	475 (2.0)	221 (2.1)	3951 (4.4)	167 (3.6)	1645 (3.5)
Either	38 953 (22.0)	186 (17.2)	3266 (13.9)	1629 (15.8)	23 806 (26.4)	844 (18.0)	9222 (19.5)

Abbreviation: MOUD, medications for opioid use disorder.

^a^ Includes American Indian or Alaska Native and Pacific Islander.

^b^ A nonfatal opioid overdose treated in an emergency department or inpatient setting; hospitalization for an injection drug use–related infection (such as acute hepatitis C, phlebitis, septic arthritis, skin or soft tissue infection, or endocarditis) combined with a diagnosis of opioid use disorder within the past 30 days; or inpatient or residential rehabilitation or detoxification care with a primary diagnosis of opioid use disorder or a diagnosis of opioid use disorder received within the previous 30 days.

### Covariates

We collected data on age, sex (female or male), race and ethnicity (ie, Asian, Black, Hispanic, White, other race [American Indian or Alaska Native and Pacific Islander], or unknown race), and insurance type (commercial, Medicaid, Medicare Advantage, other insurance). Race and ethnicity were self-reported separately (collected by insurer at the time of health plan enrollment) and then combined into mutually exclusive categories, classifying anyone who identified as Hispanic as Hispanic regardless of race; for these cases, a race response was most often missing or listed as “other,” but if there was also a race response, it was not used. Non-Hispanic individuals were categorized by race. This approach was used to ensure mutually exclusive race and ethnicity values to create the combined race and ethnicity category used in the analyses. We calculated a Charlson Comorbidity Index (CCI) score based on each beneficiary’s condition using the *International Statistical Classification of Diseases and Related Health Problems, Tenth Revision* (*ICD-10*). We also adjusted for index event year (2017-2022), state of residence, and index event type to account for temporal, geographical, and contextual variation in MOUD access.

### Statistical Analysis

Statistical analysis was conducted from October 2023 to December 2024. We performed binomial logistic regression models using the built-in generalized linear models function to examine the association of race and ethnicity and insurance type with MOUD receipt (within 180 days) for each medication, calculating adjusted odds ratios (AORs) and 95% CIs. Models were adjusted for age at index event, sex (reference: male), race and ethnicity (reference: White), insurance type (reference: commercial), index event type (reference: nonfatal opioid overdose), event year (reference: 2017), state of residence (reference: California), and CCI score. We calculated the CCI score using a 180-day lookback period instead of the standard 365 days, based on continuous insurance coverage of more than 6 months, which aligns with our inclusion criteria. This calculation was implemented using the comorbidity package in R, version 4.4.0 (R Project for Statistical Computing).^13^ In a sensitivity analysis, we found that 81.6% of individuals (150 635 of 184 662) who met the 180-day continuous insurance criterion also maintained coverage for the full 365 days, with no difference in median CCI scores or in demographics between the 2 groups, supporting the robustness of our 180-day adaptation (eTable 4 in Supplement 1). We used the emmeans package in R, version 4.4.0 to transform our AORs into estimated marginal mean values, enabling us to calculate adjusted estimated probabilities and adjusted risk differences (ARDs) in medication receipt by race and ethnicity and insurance type. The ARD indicates the absolute difference in the probability of an outcome between 2 groups after adjusting for potential confounding variables. We report both AORs and adjusted marginal estimated probabilities to enhance interpretability of the findings. eTables 2 and 3 in Supplement 1 present the unadjusted risk differences in medication receipt, which can highlight the differences in potential confounders. All analyses were conducted at the event level and performed using R, version 4.4.0.

To assess whether an interaction between race and ethnicity and insurance type was significant and improved model fit, we included an interaction term in the multivariable model and evaluated its statistical significance using the corresponding *P* value, with a 2-sided *P* < .05 considered significant. We also compared model fit with and without the interaction term using the Akaike Information Criterion. Because the interaction term was not statistically significant (*P* > .05) and did not substantially improve model fit, it was excluded from the final model for parsimony. No additional interaction terms were evaluated.

As sensitivity analyses, we (1) reestimated our primary models, excluding individuals with unknown race and ethnicity to assess the association of this heterogeneous group with our findings; (2) restricted the sample to those with continuous insurance enrollment for 180 days before and after the index event to ensure complete follow-up and to evaluate potential outcome misclassification and selection bias; and (3) reestimated our models using a shortened outcome window of 30 days instead of 180 days to assess whether observed disparities in medication receipt were evident earlier in the follow-up period. The 30-day outcome analysis also ensured that individuals were continuously enrolled in insurance at the time the outcome was measured, reducing the potential for differential loss to follow-up.

## Results

The study included 176 997 OUD-related index events involving 164 728 patients (mean [SD] patient age, 40.0 [13.1] years; 104 005 events [58.8%] involved men, and 72 992 [41.2%] involved women; 1083 events [0.6%] were among Asian patients, 23 424 [13.2%] were among Black patients, 10 302 [5.8%] were among Hispanic patients, 90 124 [50.9%] were among White patients, 4697 [2.7%] were among patients of other race and ethnicity, and 47 367 [26.8%] were among patients of unkown race) (Table 1). Most events occurred with patients with Medicaid (147 257 [83.2%]), followed by those with commercial insurance (19 179 [10.8%]), Medicare Advantage (10 192 [5.8%]), and other types of insurance (369 [0.2%]). Approximately half the events (89 577 [50.7%]) were among patients with 1 or more comorbidities; 67 002 (37.9%) had 1 to 2 comorbidities, and 22 575 (12.8%) had 3 or more comorbidities. Most index events were nonfatal opioid overdoses (125 987 [71.2%]), followed by injection drug use–related infections (32 014 [18.1%]) and rehabilitation or detoxification care (18 996 [10.7%]).

Only 34 088 events (19.3%) led to buprenorphine treatment within 180 days, and 6491 (3.7%) to naltrexone treatment within 180 days (Table 1). Buprenorphine receipt was more common among patients with Medicaid (30 240 of 147 257 [20.5%]) than those with commercial insurance (2509 of 19 179 [13.1%]), Medicare Advantage (1271 of 10 192 [12.5%]), or other insurance type (68 of 369 [18.4%]). White patients had the highest rate of buprenorphine receipt (20 880 of 90 124 events [23.2%]) compared with Black patients (2881 of 23 424 events [12.3%]), Hispanic patients (1456 of 10 302 events [14.1%]), Asian patients (161 of 1083 events [14.9%]), and patients of other races and ethnicities (706 of 4697 events [15.0%]) (Table 2). Naltrexone receipt was also highest among White patients (3951 of 90 124 events [4.4%]) and lower among other groups.

**Figure 1.**
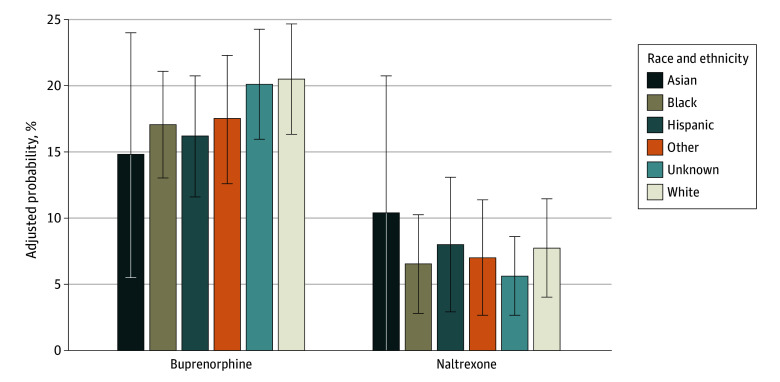
Adjusted Probabilities of Treatment With Medications for Opioid Use Disorder by Race and Ethnicity Logistic regression model adjusted for age, sex, insurance type, index event type, event year, state of residence, and Charlson Comorbidity Index score. Error bars indicate 95% CIs.

**Figure 2.**
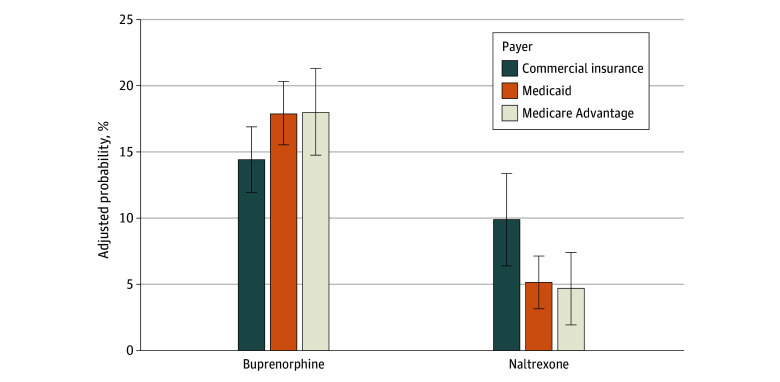
Adjusted Probabilities of Treatment With Medications for Opioid Use Disorder by Insurance Type Logistic model adjusted for age, sex, race and ethnicity, index event type, event year, state of residence, and Charlson Comorbidity Index score. Error bars indicate 95% CIs.

In adjusted models controlling for relevant covariates, Black and Hispanic patients were significantly less likely than White patients to receive buprenorphine after an opioid-related health care event. The adjusted estimated probability of receiving buprenorphine was 20.5% (95% CI, 16.4%-24.7%) for White patients, compared with 17.1% (95% CI, 13.0%-21.1%) for Black patients and 16.2% (95% CI, 11.6%-20.8%) for Hispanic patients (Black patients: AOR, 0.75 [95% CI, 0.63-0.90]; ARD, –3.4 percentage points [pp] [95% CI, –6.5 to –0.4 pp]; and Hispanic patients: AOR, 0.69 [95% CI, 0.51-0.92]; ARD, –4.4 pp [95% CI, –9.1 to –0.4 pp]) (Table 3 and Figure 1). In contrast, patients with Medicaid (AOR, 1.39 [95% CI, 1.14-1.69]; ARD, 3.5 pp [95% CI, 0.9-6.1 pp]) or Medicare Advantage (AOR, 1.40 [95% CI, 1.05-1.86]; ARD, 3.6 pp [95% CI, –0.6 to 7.7 pp]) were more likely to receive buprenorphine than those with commercial insurance (Table 3 and Figure 2).

**Table 3.** Logistic Regression of Receipt of Buprenorphine and Naltrexone 180 Days After Index

Covariates	Events, No. (%) (N = 176 997)	AOR (95% CI)^a^	
		Buprenorphine	Naltrexone
Age, mean (SD), y	40.0 (13.1)	0.98 (0.97-0.98)	0.97 (0.96-0.98)
Sex			
Female	72 992 (41.2)	0.85 (0.76-0.95)	1.35 (1.09-1.66)
Male	104 005 (58.8)	1 [Reference]	1 [Reference]
Race and ethnicity			
Asian	1083 (0.6)	0.60 (0.22-1.35)	1.47 (0.34-4.40)
Black	23 424 (13.2)	0.75 (0.63-0.90)	0.81 (0.55-1.18)
Hispanic	10 302 (5.8)	0.69 (0.51-0.92)	1.05 (0.57-1.80)
White	90 124 (50.9)	1 [Reference]	1 [Reference]
Other^b^	4697 (2.7)	0.78 (0.57-1.04)	0.88 (0.50-1.47)
Unknown	47 367 (26.8)	0.97 (0.84-1.11)	0.68 (0.51-0.90)
Index event type^c^			
Nonfatal overdose	125 987 (71.2)	1 [Reference]	1 [Reference]
Rehabilitation or detoxification care	18 996 (10.7)	2.94 (2.48-3.49)	3.44 (2.59-4.53)
Injection drug use–related infection	32 014 (18.1)	1.62 (1.42-1.85)	0.75 (0.54-1.02)
Insurance			
Commercial	19 179 (10.8)	1 [Reference]	1 [Reference]
Medicaid	147 257 (83.2)	1.39 (1.14-1.69)	0.44 (0.32-0.61)
Medicare Advantage	10 192 (5.8)	1.40 (1.05-1.86)	0.40 (0.21-0.73)
Other	369 (0.2)	1.72 (0.55-4.51)	1.08 (0.16-4.22)
Comorbidity score, mean (SD)	0.01 (0.1)	0.95 (0.85-1.06)	1.03 (0.78-1.32)
Year			
2017	25 809 (14.6)	1 [Reference]	1 [Reference]
2018	24 190 (13.7)	1.31 (1.10-1.56)	1.08 (0.78-1.48)
2019	25 204 (14.2)	1.47 (1.23-1.75)	1.10 (0.79-1.52)
2020	30 506 (17.2)	1.95 (1.65-2.32)	0.92 (0.65-1.29)
2021	36 625 (20.7)	2.05 (1.73-2.43)	0.96 (0.67-1.35)
2022	34 663 (19.6)	1.59 (1.31-1.94)	1.06 (0.71-1.54)

Abbreviation: AOR, adjusted odds ratio.

^a^ Person’s state of residence (50 states and District of Columbia) was included in the model, but not displayed in the table.

^b^ Includes American Indian or Alaska Native, and Pacific Islander.

^c^ A nonfatal opioid overdose treated in an emergency department or inpatient setting; hospitalization for an injection drug use–related infection (such as acute hepatitis C, phlebitis, septic arthritis, skin or soft tissue infection, or endocarditis) combined with a diagnosis of opioid use disorder within the past 30 days; or inpatient or residential rehabilitation or detoxification care with a primary diagnosis of opioid use disorder or a diagnosis of opioid use disorder received within the previous 30 days.

There were no statistically significant differences in receipt of naltrexone at follow-up by race and ethnicity; ARD 95% CIs crossed zero (Table 3 and Figure 1). However, patients with Medicaid (AOR, 0.44 [95% CI, 0.32-0.61]; ARD, –4.8 pp [95% CI, –7.9 to –1.6 pp]) or Medicare Advantage (AOR, 0.40 [95% CI, 0.21-0.73]; ARD, –5.2 pp [95% CI, –9.6 to –0.8 pp]) were less likely to receive naltrexone than those with commercial insurance (Table 3 and Figure 2).

The odds of buprenorphine receipt increased progressively each year from 2017 to 2021. In 2018, the adjusted odds ratio was 1.31 (95% CI, 1.10-1.56), indicating a 31% increase in the odds of buprenorphine receipt compared with 2017. By 2021, the adjusted odds ratio had increased to 2.05 (95% CI, 1.73-2.43), reflecting a 105% increase in odds relative to 2017. No significant year-over-year changes were observed in naltrexone receipt.

Results remained stable with 180-day continuous postindex enrollment (eTable 5 in Supplement 1), supporting the robustness of the findings despite potential disenrollment. Findings from the sensitivity analysis excluding individuals with unknown race and ethnicity remained consistent with the primary analysis, demonstrating similar direction and magnitude of associations across most racial and ethnic groups and insurance types (eTable 6 in Supplement 1). Slight attenuation was noted in some associations, particularly for naltrexone. In a third sensitivity analysis using a 30-day outcome window (eTable 7 in Supplement 1), effect estimates were again consistent, and disparities by race and ethnicity and insurance type were evident early in follow-up. This analysis additionally ensured insurance enrollment at the time of outcome measurement, further reinforcing the internal validity of our approach.

## Discussion

This study investigates the receipt of buprenorphine and naltrexone among a large cohort of patients after an opioid-related index event, such as overdose, inpatient rehabilitation, or drug use–related infections. Our findings reveal significant racial and ethnic and insurance-type disparities in buprenorphine receipt within 180 days of an index event. Even after adjusting for insurance type, Black and Hispanic patients were significantly less likely to receive buprenorphine compared with White patients, indicating that racial and ethnic disparities in access persist independently of insurance coverage. The use of a national multipayer database over a 5-year study period enhances the generalizability of our findings by providing a comprehensive longitudinal perspective on OUD treatment, including geographical and temporal trends. By specifically focusing on racial and ethnic differences, this study offers critical insights into MOUD access gaps and provides a foundation for targeted interventions.

Previous research using single-payer data has consistently shown racial and ethnic disparities in MOUD access. For instance, analysis of 2017-2019 Medicaid data found significantly lower odds of MOUD receipt among non-Hispanic Black, American Indian, Alaska Native, and Hispanic individuals compared with non-Hispanic White individuals, despite adjusting for clinical and demographic factors.^10^ Our study, covering a broader time range and incorporating recent policy changes—such as the elimination of the DATA 2000 (Drug Addiction Treatment Act of 2000) waiver requirement for prescribing buprenorphine and telehealth expansions during the COVID-19 pandemic—provides a more current perspective. Medicare-based studies have documented similarly significant disparities in MOUD access for Black patients, but they predated recent policies that may have influenced MOUD accessibility^9,14^ and are not generalizable to most patients who have OUD and are not covered by Medicare. Our findings suggest that despite these policy shifts, inequities in buprenorphine receipt remain pronounced among Black and Hispanic populations.

Consistent with prior research, our study found that naltrexone is prescribed less frequently than buprenorphine for OUD treatment.^9,15^ The use of extended-release naltrexone is often limited by the need for patients to complete opioid withdrawal before starting treatment and by adherence challenges.^16^ Buprenorphine, by contrast, has shown greater efficacy than extended-release naltrexone in clinical trials and observational studies, particularly in promoting abstinence and treatment retention.^17,18^ Our analysis revealed no differences in prescribing patterns of naltrexone across racial and ethnic groups—findings similar to previous research using Medicare data that did not find disparities by race and ethnicity.^9^

Disparities in buprenorphine receipt may partly reflect the unobserved receipt of methadone. Patients from racial and ethnic minority groups are disproportionately represented among patients treated with methadone, in part due to historical segregation that placed methadone clinics closer to their communities^4,19^ In contrast, predominantly White communities tend to have more buprenorphine providers per capita.^20^ Both buprenorphine and naltrexone offer treatment structures that reduce disruptions to employment and daily responsibilities while affording greater privacy and autonomy.^21,22^ These advantages highlight the need to ensure equitable access to all MOUD options.

The persistence of racial and ethnic disparities in buprenorphine access suggests that clinician-level interventions are crucial.^23,24^ Culturally sensitive care and standardized OUD screening protocols could expand access. Although the importance of culturally responsive addiction treatment is well established, there remains a critical need to expand culturally tailored strategies to MOUD delivery.^25^ Many patients report perceived bias from clinicians, which can deter treatment seeking.^22^ Health systems can help close these gaps by integrating MOUD across diverse care settings—such as primary care, emergency departments, and hospitals—and by expanding telehealth and community partnerships. These strategies are particularly valuable for patients in rural or underserved urban areas. Programs that reduce barriers to buprenorphine—such as eliminating abstinence requirements, allowing home initiation, and offering mobile induction—have shown promise in reaching patients disconnected from traditional care pathways.^26,27^

Policy changes are essential. Our findings highlight the need for public health campaigns to reduce MOUD stigma in racially and ethnically diverse communities. Medicaid’s more recent adoption of evidence-based practices—including offering all 3 forms of MOUD, using coordinated care models, and adopting harm reduction strategies—can serve as a model for other insurers. Medicaid expansions under the Patient Protection and Affordable Care Act have been associated with increased addiction treatment and reduced opioid overdose deaths, emphasizing the importance of expanding Medicaid eligibility to adults nationwide.^28,29,30^ All payers should cover the full spectrum of FDA-approved MOUD as essential health benefits, ensuring broader, equitable access.

### Limitations

Our study has several limitations. First, race and ethnicity data in insurance claims are sometime incomplete, as they are not mandatory demographic fields. This inconsistency, which varies by insurer and state, challenges analyses of racial and ethnic disparities and affects the unknown race and ethnicity category. Second, we could not assess use of methadone—an FDA-approved MOUD—due to its underreporting in pharmacy claims, limited coverage in commercial plans, and uneven geographical availability. This may have excluded important data on MOUD access, particularly for members of racial and ethnic minority groups. Although this limits full MOUD analysis, clinical evidence does not indicate race- or ethnicity-based methadone prioritization. We also included only patients with continuous insurance enrollment before and after the index event, which may limit generalizability. Our use of *ICD-10* codes based on *Diagnostic and Statistical Manual of Mental Disorders* (Fourth Edition) rather than *Diagnostic and Statistical Manual of Mental Disorders* (Fifth Edition) criteria for OUD may introduce selection bias by omitting some individuals with OUD.^31^ In addition, the high proportion of Medicaid patients reflects the known association between OUD and unemployment^32^ but may skew findings toward lower-income populations and reduce generalizability to those with other or no insurance. Although the dataset is national, it is not weighted to be nationally representative (eTable 1 in Supplement 1). We also could not determine whether patients had separate behavioral health or pharmacy coverage outside Medicaid, which varies by state. Although we adjusted for state-level differences using fixed effects, this may still introduce variability. Despite these limitations, our study provides important clinical and policy insights.

## Conclusions

In this cohort study of more than 176 000 opioid-related health care events, race and ethnicity–based and insurance-based disparities in access to buprenorphine persisted despite recent efforts to expand treatment availability. Our study highlights the need for interventions to close these gaps. Continued efforts to reduce these disparities, through both targeted clinical practices and policy reform, are essential for ensuring that all individuals have equitable access to lifesaving OUD treatments.

## Notes

Supplement 1. **eTable 1.** Characteristics of IHER Multipayor Claims Database 2017-2022 **eTable 2.** Unadjusted Risk Differences by Race/Ethnicity **eTable 3.** Unadjusted Risk Differences by Payer Type **eTable 4.** Comparison of Continuous Insurance Coverage: 180 Days vs 365 Days Pre–Index Event **eTable 5.** Comparison of Primary Analysis (30-Day Continuous Insurance Post–Index Event) and Sensitivity Analysis (180-Day Continuous Insurance Post–Index Event), 2017-2022 **eTable 6.** Comparison of Primary Analysis (Unknown Race/Ethnicity Categorized) and Sensitivity Analysis (Unknown Race/Ethnicity Excluded), 2017-2022 **eTable 7.** Comparison of Primary Analysis (180-Day Post–Index Event Outcome) and Sensitivity Analysis (30-Day Post–Index Event Outcome), 2017-2022 **eFigure.** Study Inclusion and Exclusion Flowchart: Retrospective Analysis

Supplement 2.
Data Sharing Statement
