# Error in Abstract

**DOI:** 10.1001/jamanetworkopen.2025.27830

**Published:** 2025-07-24

In the Original Investigation titled “Receipt of Buprenorphine and Naltrexone for Opioid Use Disorder by Race and Ethnicity and Insurance Type,”^1^ published June 26, 2025, the number of opioid-related index health care events was incorrectly stated as more than 17 600 in the abstract; the correct number is 176 000. This article has been corrected.

## References

[1] Khatri UG, Lopez C, Yen YT, Ling EJ, Richardson LD, Ngai KM. Receipt of buprenorphine and naltrexone for opioid use disorder by race and ethnicity and insurance type. JAMA Netw Open. 2025;8(6):e2518493. doi:10.1001/jamanetworkopen.2025.18493 40569592 PMC12203278

