# Supplementary material for: Receipt of Buprenorphine and Naltrexone for Opioid Use Disorder by Race and Ethnicity and Insurance Type
Source: JAMA Netw Open. 2025 Jun 26;8(6):e2518493. doi: 10.1001/jamanetworkopen.2025.18493 (PMC12203278; doi:10.1001/jamanetworkopen.2025.18493)
Supplement: Supplement 1. — eTable 1. Characteristics of IHER Multipayor Claims Database 2017-2022 eTable 2. Unadjusted Risk Differences by Race/Ethnicity eTable 3. Unadjusted Risk Differences by Payer Type eTable 4. Comparison of Continuous Insurance Coverage: 180 Days vs 365 Days Pre–Index Event eTable 5. Comparison of Primary Analysis (30-Day Continuous Insurance Post–Index Event) and Sensitivity Analysis (180-Day Continuous Insurance Post–Index Event), 2017-2022 eTable 6. Comparison of Primary Analysis (Unknown Race/Ethnicity Categorized) and Sensitivity Analysis (Unknown Race/Ethnicity Excluded), 2017-2022 eTable 7. Comparison of Primary Analysis (180-Day Post–Index Event Outcome) and Sensitivity Analysis (30-Day Post–Index Event Outcome), 2017-2022 eFigure. Study Inclusion and Exclusion Flowchart: Retrospective Analysis [file jamanetwopen-e2518493-s001.pdf]

# Supplementary Online Content

Khatri UG, Lopez C, Yen YT, Ling EJ, Richardson LD, Ngai KM. Receipt of buprenorphine and naltrexone for opioid use disorder by race and ethnicity and insurance type. *JAMA Netw Open.* 2025;8(6):e2518493. doi:10.1001/jamanetworkopen.2025.18493

**eTable 1.** Characteristics of IHER Multipayor Claims Database 2017-2022

**eTable 2.** Unadjusted Risk Differences by Race/Ethnicity

**eTable 3.** Unadjusted Risk Differences by Payer Type

**eTable 4.** Comparison of Continuous Insurance Coverage: 180 Days vs 365 Days Pre–Index Event

**eTable 5.** Comparison of Primary Analysis (30-Day Continuous Insurance Post–Index Event) and Sensitivity Analysis (180-Day Continuous Insurance Post–Index Event), 2017-2022

**eTable 6.** Comparison of Primary Analysis (Unknown Race/Ethnicity Categorized) and Sensitivity Analysis (Unknown Race/Ethnicity Excluded), 2017-2022

**eTable 7.** Comparison of Primary Analysis (180-Day Post–Index Event Outcome) and Sensitivity Analysis (30-Day Post–Index Event Outcome), 2017-2022

**eFigure.** Study Inclusion and Exclusion Flowchart: Retrospective Analysis

This supplementary material has been provided by the authors to give readers additional information about their work.

© 2025 Khatri UG et al. *JAMA Network Open.*

**eTable 1.** Characteristics of IHER Multipayor Claims Database 2017-2022

| Characteristic | Events, No. (%) |
|---|---|
| **Age Group** | |
| <18 | 3025853293 (14.3) |
| 18-24 | 1266354874 (6.0) |
| 25-34 | 2241526225 (10.6) |
| 35-44 | 2389294862 (11.3) |
| 45-64 | 7377199151 (34.9) |
| 65+ | 4831715895 (22.9) |
| **Sex** | |
| Female | 12227615736 (58.7) |
| Male | 8610944398 (41.3) |
| **Race/Ethnicity** | |
| Asian | 558062651 (2.7) |
| Black | 2500276684 (12.0) |
| Hispanic | 1933368859 (9.3) |
| White | 6848060808 (32.9) |
| Other[a] | 752174225 (3.6) |
| Unknown | 8246616907 (39.6) |
| **Insurance** | |
| Commercial | 7227478720 (34.2) |
| Medicaid | 8829417909 (41.8) |
| Medicare Advantage | 3309379039 (15.7) |
| Other | 1765668632 (8.4) |

a. Includes Native American/Alaskan Native, Pacific Islander

© 2025 Khatri UG et al. *JAMA Network Open.*

**eTable 2.** Unadjusted Risk Differences by Race/Ethnicity

| Race/Ethnicity | Medication Type | Proportion (%) | Unadjusted Risk Difference |
|---|---|---|---|
| **Asian** | Buprenorphine | 14.9 | -8.2 (-19.0, 2.6) |
| **Black** | Buprenorphine | 12.3 | -10.8 (-21.3, -0.3) |
| **Hispanic** | Buprenorphine | 14.1 | -9.0 (-19.7, 1.7) |
| **Other**[a] | Buprenorphine | 15.0 | -8.1 (-18.9, 2.7) |
| **Unknown** | Buprenorphine | 16.9 | -6.2 (-17.3, 4.9) |
| **White** | Buprenorphine | 23.2 | REF |
| **Asian** | Naltrexone | 3.0 | -1.4 (-6.6, 3.8) |
| **Black** | Naltrexone | 2.0 | -2.4 (-7.3, 2.5) |
| **Hispanic** | Naltrexone | 2.1 | -2.3 (-7.2, 2.6) |
| **Other**[a] | Naltrexone | 3.6 | -0.8 (-6.2, 4.6) |
| **Unknown** | Naltrexone | 3.5 | -0.9 (-6.3, 4.5) |
| **White** | Naltrexone | 4.4 | REF |

a. Includes Native American/Alaskan Native, Pacific Islander

© 2025 Khatri UG et al. *JAMA Network Open.*

**eTable 3.** Unadjusted Risk Differences by Payer Type

| Payer | Medication | Proportion (%) | Unadjusted Risk Difference |
|---|---|---|---|
| **Medicaid** | Buprenorphine | 20.5 | 7.4 (-2.9, 17.7) |
| **Medicare Adv** | Buprenorphine | 12.5 | -0.6 (-9.9, 8.7) |
| **Commercial** | Buprenorphine | 13.1 | REF |
| **Medicaid** | Naltrexone | 3.6 | -1.6 (-7.3, 4.1) |
| **Medicare Adv** | Naltrexone | 1.8 | -3.4 (-8.5, 1.7) |
| **Commercial** | Naltrexone | 5.2 | REF |

© 2025 Khatri UG et al. *JAMA Network Open.*

**eTable 4.** Comparison of Continuous Insurance Coverage: 180 Days vs 365 Days Pre–Index Event

| **Characteristic** | **Events, No. (%) (N=359596)** | **180 days (N=198747)** | **365 days (N=160849)** | **Cramer's V Effect Size** |
|---|---|---|---|---|
| **Age Group** | | | | 0.02 |
| 18-24 | 33169 (9.2) | 18194 (9.2) | 14975 (9.3) | |
| 25-34 | 106359 (29.6) | 60209 (30.3) | 46150 (28.7) | |
| 35-44 | 87356 (24.3) | 48669 (24.5) | 38687 (24.1) | |
| 45-64 | 116764 (32.5) | 63168 (31.8) | 53596 (33.3) | |
| 65+ | 15948 (4.4) | 8507 (4.3) | 7441 (4.6) | |
| **Sex** | | | | 0.01 |
| Female | 150155 (41.8) | 82034 (41.3) | 68121 (42.4) | |
| Male | 209441 (58.2) | 116713 (58.7) | 92728 (57.6) | |
| **Race** | | | | 0.01 |
| Asian | 2175 (0.6) | 1214 (0.6) | 961 (0.6) | |
| Black | 48998 (13.6) | 26669 (13.4) | 22329 (13.9) | |
| Hispanic | 21350 (5.9) | 11733 (5.9) | 9617 (6.0) | |
| White | 180158 (50.1) | 100091 (50.4) | 80067 (49.8) | |
| Other[a] | 9943 (2.8) | 5444 (2.7) | 4499 (2.8) | |
| Unknown | 96972 (27.0) | 53596 (27.0) | 43376 (27.0) | |
| **Index Event Type[b]** | | | | 0.00 |
| NF Overdose | 253826 (70.6) | 139989 (70.4) | 113837 (70.8) | |
| Rehab/Detox | 36076 (10.0) | 19982 (10.1) | 16094 (10.0) | |
| IDU Infection | 69694 (19.4) | 38776 (19.5) | 30918 (19.2) | |
| **Payer** | | | | 0.01 |
| Commercial | 39694 (11.0) | 21402 (10.8) | 18292 (11.4) | |
| Medicaid | 295196 (82.1) | 163825 (82.4) | 131371 (81.7) | |
| Medicare Advantage | 23630 (6.6) | 12909 (6.5) | 10721 (6.7) | |
| Other | 1076 (0.3) | 611 (0.3) | 465 (0.3% | |

a. Includes Native American/Alaskan Native, Pacific Islander; b.1) A nonfatal opioid overdose treated in an emergency department or inpatient setting; 2) hospitalization for an injection drug use-related infection (such as acute hepatitis C, phlebitis, septic arthritis, skin or soft tissue infection, or endocarditis) combined with a diagnosis of opioid use disorder (OUD) within the past 30 days; or 3) inpatient or residential rehabilitation or detoxification care with a primary diagnosis of OUD or a diagnosis of OUD received within the previous 30 days

© 2025 Khatri UG et al. *JAMA Network Open.*

**eTable 5.** Comparison of Primary Analysis (30-Day Continuous Insurance Post–Index Event) and Sensitivity Analysis (180-Day Continuous Insurance Post–Index Event), 2017-2022

| Covariates | Primary Analysis | | | 180-Day of Continuous Insurance Post-Index Event [a] | | |
|---|---|---|---|---|---|---|
| | | Buprenorphine | Naltrexone | | Buprenorphine | Naltrexone |
| | Events, No. (%) (N=176997) | aOR (95% CI) | aOR (95% CI) | Events, No. (%) (N=152008) | aOR (95% CI) | aOR (95% CI |
| **Age, mean (SD)** | 40.0 (13.1) | 0.98 (0.97, 0.98) | 0.97 (0.96, 0.98) | 40.1 (13.1) | 0.97 (0.97, 0.98) | 0.97 (0.96, 0.98) |
| **Sex** | | | | | | |
| Male | 72992 (41.2) | — | — | 88635 (58.3) | — | — |
| Female | 104005 (58.8) | 0.85 (0.76, 0.95) | 1.35 (1.09, 1.66) | 63373 (41.7) | 0.84 (0.75, 0.95) | 1.22 (0.96, 1.55) |
| **Race/Ethnicity** | | | | | | |
| White | 90124 (50.9) | — | — | 77526 (51.0) | — | — |
| Asian | 1083 (0.6) | 0.60 (0.22, 1.35) | 1.47 (0.34, 4.40) | 920 (0.6) | 0.48 (0.14, 1.28) | 1.05 (0.16, 3.92) |
| Black | 23424 (13.2) | 0.75 (0.63, 0.90) | 0.81 (0.55, 1.18) | 20643 (13.6) | 0.74 (0.61, 0.90) | 0.97 (0.65, 1.43) |
| Hispanic | 10302 (5.8) | 0.69 (0.51, 0.92) | 1.05 (0.57, 1.80) | 9001 (5.9) | 0.69 (0.49, 0.95) | 1.06 (0.54, 1.91) |
| Other[b] | 4697 (2.7) | 0.78 (0.57, 1.04) | 0.88 (0.50, 1.47) | 3879 (2.6) | 0.77 (0.54, 1.09) | 0.71 (0.34, 1.33) |
| Unknown | 47367 (26.8) | 0.97 (0.84, 1.11) | 0.68 (0.51, 0.90) | 40039 (26.3) | 1.00 (0.86, 1.17) | 0.64 (0.46, 0.89) |
| **Index Event Type[c]** | | | | | | |
| Non-Fatal Overdose | 125987 (71.2) | — | — | 108246 (71.2) | — | — |
| Rehab/Detox | 18996 (10.7) | 2.94 (2.48, 3.49) | 3.44 (2.59, 4.53) | 16874 (11.1) | 2.87 (2.37, 3.47) | 3.11 (2.26, 4.25) |
| IDU Infection | 32014 (18.1) | 1.62 (1.42, 1.85) | 0.75 (0.54, 1.02) | 26888 (17.7) | 1.67 (1.44, 1.95) | 0.74 (0.50, 1.06) |
| **Insurance** | | | | | | |
| Commercial | 19179 (10.8) | — | — | 15561 (10.2) | — | — |
| Medicaid | 147257 (83.2) | 1.39 (1.14, 1.69) | 0.44 (0.32, 0.61) | 127674 (84.0) | 1.40 (1.13, 1.74) | 0.43 (0.30, 0.62) |
| Medicare Advantage | 10192 (5.8) | 1.40 (1.05, 1.86) | 0.40 (0.21, 0.73) | 8474 (5.6) | 1.48 (1.06, 2.05) | 0.29 (0.13, 0.61) |
| Other | 369 (0.2) | 1.72 (0.55, 4.51) | 1.08 (0.16, 4.22) | 299 (0.2) | 2.28 (0.58, 7.30) | 1.42 (0.20, 6.05) |
| **Comorbidity Score, mean (SD)** | 0.01 (0.1) | 0.95 (0.85, 1.06) | 1.03 (0.78, 1.32) | 0.00728 (0.120) | 0.92 (0.81, 1.04) | 1.03 (0.74, 1.38) |
| **Year** | | | | | | |
| 2017 | 25809 (14.6) | — | — | 20340 (13.4) | — | — |
| 2018 | 24190 (13.7) | 1.31 (1.10, 1.56) | 1.08 (0.78, 1.48) | 19005 (12.5) | 1.26 (1.03, 1.55) | 1.12 (0.77, 1.61) |
| 2019 | 25204 (14.2) | 1.47 (1.23, 1.75) | 1.10 (0.79, 1.52) | 19891 (13.1) | 1.44 (1.17, 1.77) | 0.95 (0.64, 1.41) |
| 2020 | 30506 (17.2) | 1.95 (1.65, 2.32) | 0.92 (0.65, 1.29) | 27984 (18.4) | 1.85 (1.53, 2.24) | 0.95 (0.65, 1.37) |
| 2021 | 36625 (20.7) | 2.05 (1.73, 2.43) | 0.96 (0.67, 1.35) | 33482 (22.0) | 2.03 (1.67, 2.46) | 0.99 (0.68, 1.45) |
| 2022 | 34663 (19.6) | 1.59 (1.31, 1.94) | 1.06 (0.71, 1.54) | 31306 (20.6) | 1.60 (1.27, 2.00) | 1.99 (0.63, 1.53) |

a. Person's state of resident (50 states and District of Columbia) was included in the model, but not displayed in the table; b. Includes Native American/Alaskan Native, Pacific Islander; c. 1) A nonfatal opioid overdose treated in an emergency department or inpatient setting; 2) hospitalization for an injection drug use-related infection (such as acute hepatitis C, phlebitis, septic arthritis, skin or soft tissue infection, or endocarditis) combined with a diagnosis of opioid use disorder (OUD) within the past 30 days; or 3) inpatient or residential rehabilitation or detoxification care with a primary diagnosis of OUD or a diagnosis of OUD received within the previous 30 days

© 2025 Khatri UG et al. *JAMA Network Open.*

**eTable 6.** Comparison of Primary Analysis (Unknown Race/Ethnicity Categorized) and Sensitivity Analysis (Unknown Race/Ethnicity Excluded), 2017-2022

| Covariates | Primary Analysis[a] | | | Unknown Race/Ethnicity Excluded[a] | | |
|---|---|---|---|---|---|---|
| | | Buprenorphine | Naltrexone | | Buprenorphine | Naltrexone |
| | Events, No. (%) (N=176997) | aOR (95% CI) | aOR (95% CI) | Events, No. (%) (N=129630) | aOR (95% CI) | aOR (95% CI |
| **Age, mean (SD)** | 40.0 (13.1) | 0.98 (0.97, 0.98) | 0.97 (0.96, 0.98) | 40.0 (12.9) | 0.98 (0.97, 0.98) | 0.97 (0.96, 0.98) |
| **Sex** | | | | | | |
| Male | 72992 (41.2) | — | — | 75142 (58.0) | — | — |
| Female | 104005 (58.8) | 0.85 (0.76, 0.95) | 1.35 (1.09, 1.66) | 54488 (42.0) | 0.86 (0.76, 0.97) | 1.28 (1.01, 1.62) |
| **Race/Ethnicity** | | | | | | |
| White | 90124 (50.9) | — | — | 90124 (69.5) | — | — |
| Asian | 1083 (0.6) | 0.60 (0.22, 1.35) | 1.47 (0.34, 4.40) | 1083 (0.8) | 0.62 (0.23, 1.41) | 1.67 (0.38, 4.98) |
| Black | 23424 (13.2) | 0.75 (0.63, 0.90) | 0.81 (0.55, 1.18) | 23424 (18.1) | 0.74 (0.62, 0.89) | 0.84 (0.56, 1.22) |
| Hispanic | 10302 (5.8) | 0.69 (0.51, 0.92) | 1.05 (0.57, 1.80) | 10302 (7.9) | 0.69 (0.51, 0.92) | 0.96 (0.52, 1.66) |
| Other[b] | 4697 (2.7) | 0.78 (0.57, 1.04) | 0.88 (0.50, 1.47) | 4697 (3.6) | 0.79 (0.58, 1.07) | 0.89 (0.50, 1.49) |
| Unknown | 47367 (26.8) | 0.97 (0.84, 1.11) | 0.68 (0.51, 0.90) | — | — | — |
| **Index Event Type[c]** | | | | | | |
| Non-Fatal Overdose | 125987 (71.2) | — | — | 91027 (70.2) | — | — |
| Rehab/Detox | 18996 (10.7) | 2.94 (2.48, 3.49) | 3.44 (2.59, 4.53) | 14839 (11.4) | 3.34 (2.76, 4.04) | 3.66 (2.66, 5.02) |
| IDU Infection | 32014 (18.1) | 1.62 (1.42, 1.85) | 0.75 (0.54, 1.02) | 23764 (18.3) | 1.59 (1.37, 1.86) | 0.90 (0.62, 1.27) |
| **Insurance** | | | | | | |
| Commercial | 19179 (10.8) | — | — | 7977 (6.2) | — | — |
| Medicaid | 147257 (83.2) | 1.39 (1.14, 1.69) | 0.44 (0.32, 0.61) | 114395 (88.2) | 1.39 (1.06, 1.86) | 0.41 (0.28, 0.63) |
| Medicare Advantage | 10192 (5.8) | 1.40 (1.05, 1.86) | 0.40 (0.21, 0.73) | 7039 (5.4) | 1.35 (0.92, 1.98) | 0.42 (0.20, 0.84) |
| Other | 369 (0.2) | 1.72 (0.55, 4.51) | 1.08 (0.16, 4.22) | 219 (0.2) | 2.87 (0.73, 9.38) | 1.88 (0.26, 8.41) |
| **Comorbidity Score, mean (SD)** | 0.01 (0.1) | 0.95 (0.85, 1.06) | 1.03 (0.78, 1.32) | 0.0121 (0.155) | 0.95 (0.84, 1.06) | 0.95 (0.72, 1.24) |
| **Year** | | | | | | |
| 2017 | 25809 (14.6) | — | — | 18486 (14.3) | — | — |
| 2018 | 24190 (13.7) | 1.31 (1.10, 1.56) | 1.08 (0.78, 1.48) | 17163 (13.2) | 1.34 (1.09, 1.65) | 0.84 (0.57, 1.22) |
| 2019 | 25204 (14.2) | 1.47 (1.23, 1.75) | 1.10 (0.79, 1.52) | 17915 (13.8) | 1.54 (1.25, 1.89) | 0.95 (0.65, 1.38) |
| 2020 | 30506 (17.2) | 1.95 (1.65, 2.32) | 0.92 (0.65, 1.29) | 22688 (17.5) | 1.97 (1.62, 2.40) | 0.81 (0.55, 1.18) |
| 2021 | 36625 (20.7) | 2.05 (1.73, 2.43) | 0.96 (0.67, 1.35) | 27338 (21.1) | 2.05 (1.68, 2.49) | 0.85 (0.57, 1.24) |
| 2022 | 34663 (19.6) | 1.59 (1.31, 1.94) | 1.06 (0.71, 1.54) | 26040 (20.1) | 1.66 (1.33, 2.08) | 0.90 (0.58, 1.38) |

a. Person's state of resident (50 states and District of Columbia) was included in the model, but not displayed in the table; b. Includes Native American/Alaskan Native, Pacific Islander; c. 1) A nonfatal opioid overdose treated in an emergency department or inpatient setting; 2) hospitalization for an injection drug use-related infection (such as acute hepatitis C, phlebitis, septic arthritis, skin or soft tissue infection, or endocarditis) combined with a diagnosis of opioid use disorder (OUD) within the past 30 days; or 3) inpatient or residential rehabilitation or detoxification care with a primary diagnosis of OUD or a diagnosis of OUD received within the previous 30 days

© 2025 Khatri UG et al. *JAMA Network Open.*

**eTable 7.** Comparison of Primary Analysis (180-Day Post–Index Event Outcome) and Sensitivity Analysis (30-Day Post–Index Event Outcome), 2017-2022

| Covariates | Events, No. (%) (N=176997) | Primary Analysis[a,b] Buprenorphine aOR (95% CI) | Primary Analysis[a,b] Naltrexone aOR (95% CI) | 30-Day Post-Index Event Outcome[a,b] Buprenorphine aOR (95% CI) | 30-Day Post-Index Event Outcome[a,b] Naltrexone aOR (95% CI |
|---|---|---|---|---|---|
| **Age, mean (SD)** | 40.0 (13.1) | 0.98 (0.97, 0.98) | 0.97 (0.96, 0.98) | 0.98 (0.97, 0.99) | 0.96 (0.95, 0.98) |
| **Sex** | | | | | |
| Male | 72992 (41.2) | — | — | — | — |
| Female | 104005 (58.8) | 0.85 (0.76, 0.95) | 1.35 (1.09, 1.66) | 0.85 (0.74, 0.97) | 1.55 (1.11, 2.16) |
| **Race/Ethnicity** | | | | | |
| White | 90124 (50.9) | — | — | — | — |
| Asian | 1083 (0.6) | 0.60 (0.22, 1.35) | 1.47 (0.34, 4.40) | 0.56 (0.13, 1.61) | 0.83 (0.04, 4.82) |
| Black | 23424 (13.2) | 0.75 (0.63, 0.90) | 0.81 (0.55, 1.18) | 0.73 (0.58, 0.92) | 0.55 (0.25, 1.07) |
| Hispanic | 10302 (5.8) | 0.69 (0.51, 0.92) | 1.05 (0.57, 1.80) | 0.62 (0.41, 0.91) | 1.26 (0.47, 2.78) |
| Other[c] | 4697 (2.7) | 0.78 (0.57, 1.04) | 0.88 (0.50, 1.47) | 0.82 (0.54, 1.20) | 1.06 (0.42, 2.31) |
| Unknown | 47367 (26.8) | 0.97 (0.84, 1.11) | 0.68 (0.51, 0.90) | 1.14 (0.96, 1.35) | 0.63 (0.38, 1.00) |
| **Index Event Type[d]** | | | | | |
| Non-Fatal Overdose | 125987 (71.2) | — | — | — | — |
| Rehab/Detox | 18996 (10.7) | 2.94 (2.48, 3.49) | 3.44 (2.59, 4.53) | 4.12 (3.40, 5.00) | 6.36 (4.21, 9.60) |
| IDU Infection | 32014 (18.1) | 1.62 (1.42, 1.85) | 0.75 (0.54, 1.02) | 1.44 (1.21, 1.70) | 0.56 (0.30, 0.98) |
| **Insurance** | | | | | |
| Commercial | 19179 (10.8) | — | — | — | — |
| Medicaid | 147257 (83.2) | 1.39 (1.14, 1.69) | 0.44 (0.32, 0.61) | 1.47 (1.14, 1.90) | 0.49 (0.29, 0.84) |
| Medicare Advantage | 10192 (5.8) | 1.40 (1.05, 1.86) | 0.40 (0.21, 0.73) | 1.58 (1.09, 2.27) | 0.36 (0.12, 0.97) |
| Other | 369 (0.2) | 1.72 (0.55, 4.51) | 1.08 (0.16, 4.22) | 2.64 (0.71, 7.72) | 1.14 (0.06, 6.96) |
| **Comorbidity Score, mean (SD)** | 0.01 (0.1) | 0.95 (0.85, 1.06) | 1.03 (0.78, 1.32) | 1.06 (0.92, 1.21) | 1.02 (0.62, 1.54) |
| **Year** | | | | | |
| 2017 | 25809 (14.6) | — | — | — | — |
| 2018 | 24190 (13.7) | 1.31 (1.10, 1.56) | 1.08 (0.78, 1.48) | 1.45 (1.14, 1.84) | 0.99 (0.58, 1.67) |
| 2019 | 25204 (14.2) | 1.47 (1.23, 1.75) | 1.10 (0.79, 1.52) | 1.68 (1.32, 2.14) | 1.08 (0.63, 1.82) |
| 2020 | 30506 (17.2) | 1.95 (1.65, 2.32) | 0.92 (0.65, 1.29) | 2.52 (2.01, 3.17) | 0.70 (0.39, 1.24) |
| 2021 | 36625 (20.7) | 2.05 (1.73, 2.43) | 0.96 (0.67, 1.35) | 2.53 (2.02, 3.18) | 1.08 (0.63, 1.84) |
| 2022 | 34663 (19.6) | 1.59 (1.31, 1.94) | 1.06 (0.71, 1.54) | 2.12 (1.64, 2.73) | 1.01 (0.54, 1.84) |

a. Both analyses includes 30-day continuous insurance post-index event, b. Person's state of resident (50 states and District of Columbia) was included in the model, but not included in the table; c. Includes Native American/Alaskan Native, Pacific Islander; d.1) A nonfatal opioid overdose treated in an emergency department or inpatient setting; 2) hospitalization for an injection drug use-related infection (such as acute hepatitis C, phlebitis, septic arthritis, skin or soft tissue infection, or endocarditis) combined with a diagnosis of opioid use disorder (OUD) within the past 30 days; or 3) inpatient or residential rehabilitation or detoxification care with a primary diagnosis of OUD or a diagnosis of OUD received within the previous 30 days.

© 2025 Khatri UG et al. *JAMA Network Open.*

**eFigure.** Study Inclusion and Exclusion Flowchart: Retrospective Analysis

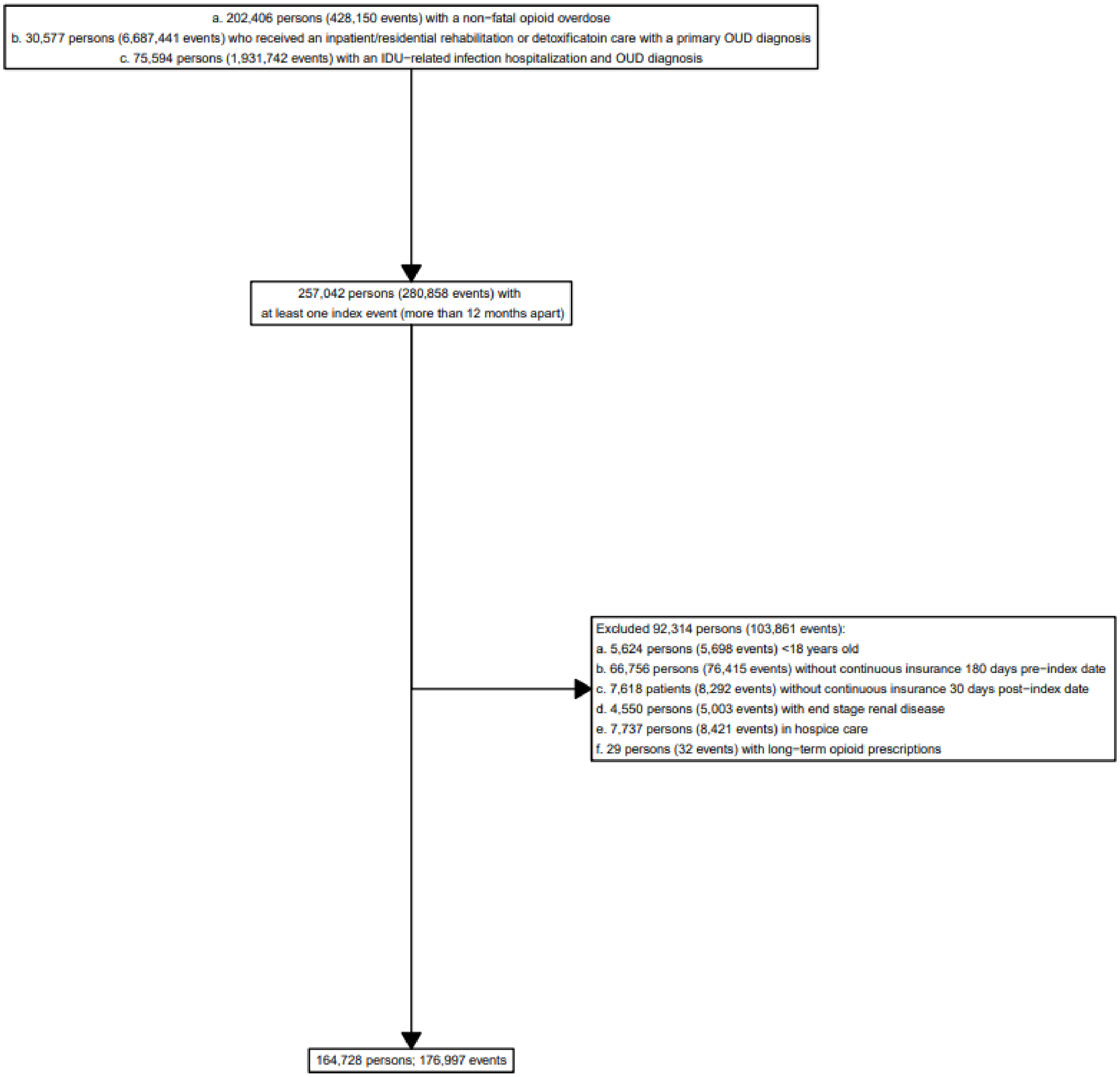


© 2025 Khatri UG et al. *JAMA Network Open.*
