# Supplementary material for: Receipt of Buprenorphine and Naltrexone for Opioid Use Disorder by Race and Ethnicity and Insurance Type
Source: JAMA Netw Open. 2025 Jun 26;8(6):e2518493. doi: 10.1001/jamanetworkopen.2025.18493 (PMC12203278; doi:10.1001/jamanetworkopen.2025.18493)
Supplement: Supplement 2. — Data Sharing Statement [file jamanetwopen-e2518493-s002.pdf]

Data Sharing Statement

Khatri. Receipt of Buprenorphine and Naltrexone for Opioid Use Disorder by Race and Ethnicity and Insurance Type. *JAMA Netw Open*. Published June 26, 2025.
doi:10.1001/jamanetworkopen.2025.18493

Data
Data available: No
